# Supplementary figures and images for: Effects of Diethyl Phosphate, a Non-Specific Metabolite of Organophosphorus Pesticides, on Serum Lipid, Hormones, Inflammation, and Gut Microbiota
Source: Molecules. 2019 May 24;24(10):2003. doi: 10.3390/molecules24102003 (PMC6572208; doi:10.3390/molecules24102003)

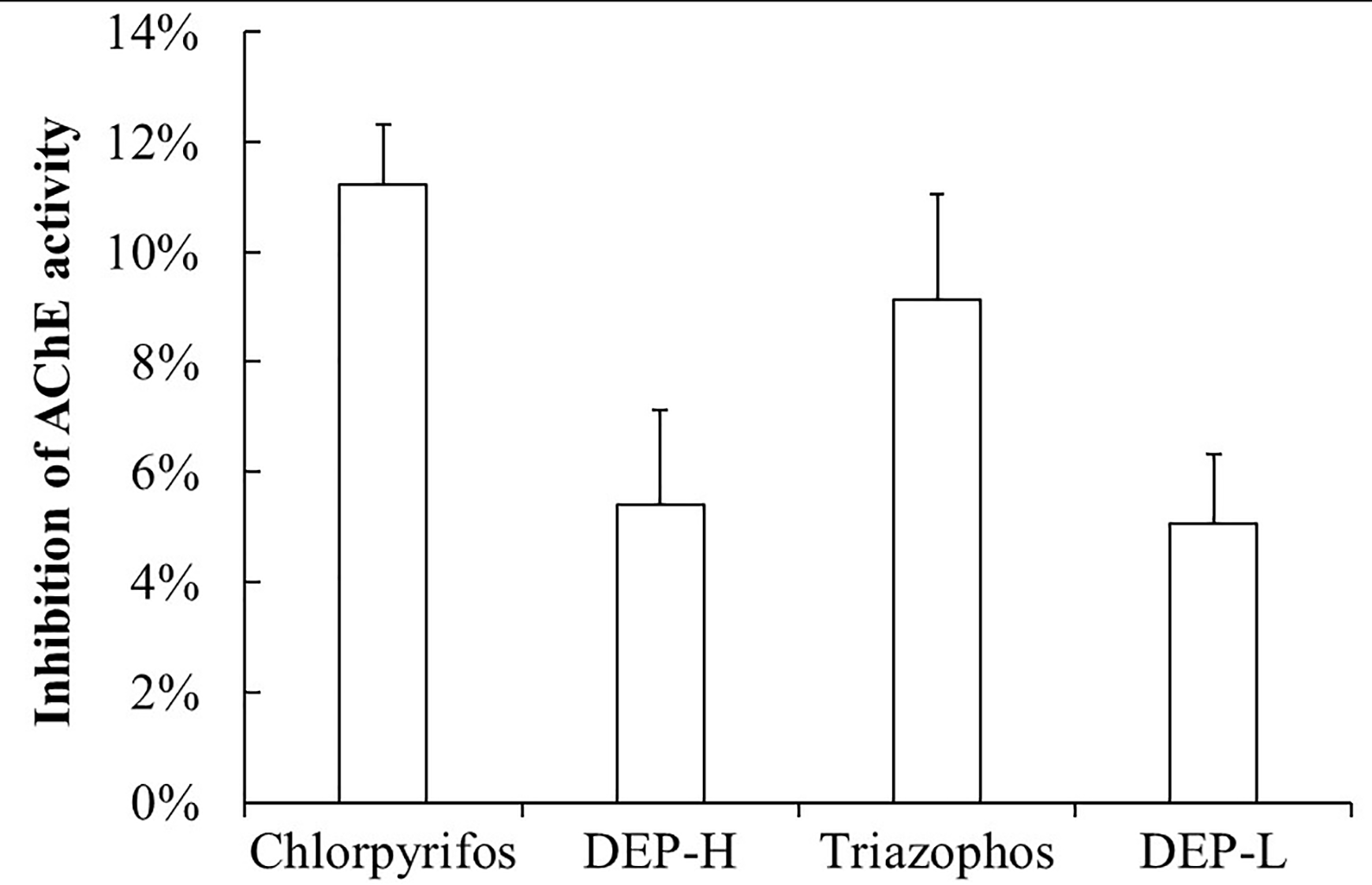

Supplement: Supplementary file 1 [file molecules-24-02003-s001.zip › molecules-508396-supplementary.tif]
